# Renal function at 12 months of kidney transplantation comparing tacrolimus and mycophenolate with tacrolimus and mTORi in donors with different KDPI ranges. A multicenter cohort study using propensity scoring

**DOI:** 10.3389/frtra.2023.1279940

**Published:** 2023-10-16

**Authors:** Arlisson Macedo Rodrigues, Mariana Tavares Tanno, Mariana Moraes Contti, Hong Si Nga, Mariana Farina Valiatti, Silvana Daher Costa, Tainá Veras de Sandes-Freitas, Ronaldo de Matos Esmeraldo, Camila Marinho Assunção, Juliana Bastos Campos Tassi, Gustavo Fernandes Ferreira, Claudia Rosso Felipe, Jose Osmar Medina Pestana, Helio Tedesco Silva, Luis Gustavo Modelli de Andrade

**Affiliations:** ^1^ Division of Nephrology, Department of Internal Medicine, Universidade Estadual Paulista (UNESP), Botucatu, Brazil; ^2^ Transplant Unit, Hospital Geral de Fortaleza, Fortaleza, Brazil; ^3^ Transplant Unit, Santa Casa de Misericórdia de Juiz de Fora, Juiz de Fora, Brazil; ^4^ Division of Nephrology, Universidade Federal de São Paulo, Hospital do Rim, São Paulo, Brazil

**Keywords:** kidney transplantation, immunosuppression, mTORi, mycophenolate, KDPI

## Abstract

**Introduction:**

The combination of tacrolimus/mTORi compared to tacrolimus/mycophenolate (MMF) was shown to be safe in the TRANSFORM trial. For donors with a high KDPI (Kidney Donor Profile Index), however, there are no data to support the effectiveness of this regimen. The main objective of this study was to explore the influence of the KDPI on 12-month renal function (eGFR) in patients receiving mTORi or MMF.

**Methods:**

Multicenter cohort study of four Brazilian services that use the tacrolimus with mTORi as a protocol. Data from 2008 to 2018 of the tacrolimus/mycophenolate (MMF) and tacrolimus/mTORi (mTORi) regimens in renal transplant recipients over 18 years old were collected. For better homogeneity, the propensity score was used. Afterward, the method used for group selection (“match”) was the K-nearest neighbor (KNN) method. New analyses were performed on this new balanced sample, and two different subsamples were constituted based on the median KDPI.

**Results:**

The global analysis (*n* = 870) showed that the major determinant of worse kidney function was high KDPI. Afterward, the three strata were analyzed. In the first stratum (KDPI up to 50), 242 patients were evaluated, with 121 in each group. The eGFR was 64 ml/min/1.73 m2 in the mTORi group compared to 63 in the MMF group, *p* = 0.4, and when imputed eGFR was evaluated, 61 in the mTORi and 53 in the MMF, *p* = 0.065. In the second stratum (KDPI from 50 to 85), 282 patients were evaluated, with 141 in each group. eGFR was 46 ml/min/1.73 m2 in mTORi compared to 48 in MMF, *p* = 0.4, and when imputed eGFR was evaluated, 40 mTORi and 41 MMF, *p* = 0.8. In the last stratum (KDPI higher than 85) with *n* = 126 and 63 cases per group, eGFR was 36 ml/min/1.73 m2 in mTORi compared to 39 in MMF, *p* = 0.2, and when imputed eGFR was evaluated, 30 mTORi and 34 MMF, *p* = 0.2.

**Discussion:**

The regimen using mTOR inhibitor is an effective and safe regimen when compared to the standard regimen. In addition, the scheme seems to offer additional protection against infections and may be an important ally in cases of high risk for these pathologies.

## Introduction

Chronic kidney disease (CKD) is a condition related to an increased risk of hospitalization, morbidity, and mortality from cardiovascular disease that gradually progresses to end-stage disease, which requires renal replacement therapy with dialysis or kidney transplantation (1). When eligible, transplantation is considered the best treatment for these patients, enabling higher survival and better quality of life (2).

The central issue regarding organ transplantation remains the suppression of allograft rejection. Thus, developing immunosuppressive drugs is the key to successful kidney transplantation (3). The currently considered standard immunosuppression regimen consists of the combination of tacrolimus associated with mycophenolate and prednisone, with renal graft survival rates greater than 90% at one year and an incidence of acute rejection lower than 15% (4).

A proposal for alternative immunosuppression to the standard regimen is the combination of a calcineurin inhibitor at low doses associated with a mTORi (mammalian target of rapamycin inhibitor) with prednisone (5, 6). This regimen was tested in a large clinical trial comparing calcineurin inhibitor associated with mycophenolate to calcineurin inhibitor associated with mTORi, demonstrating similar results for renal function, acute rejection rates, and graft survival (7). A possible advantage of this combination is the proven reduction in the incidence of cytomegalovirus (8, 9). This finding is particularly useful in developing countries where the costs of pharmacological prophylaxis or preemptive therapy represent a significant portion of the costs associated with kidney transplantation. Valiatti, in a drug economy study, demonstrated a cost reduction in the mTORi group in the order of R$ 4,500 to R$ 6,200 in favor of this group (10), a result similar to that found by Felipe et al. in which regimens containing mTORi are more cost-effective (11).

Based on this information, some Brazilian centers started using the tacrolimus regimen associated with mTORi to reduce the incidence of cytomegalovirus while maintaining the same efficacy (12). In recent years, however, we have observed an increase in the number of kidney transplants with expanded criteria donors (donor aged ≥60 years or ≥50 years with 2 of the following conditions: history of arterial hypertension, serum creatinine ≥1.5 mg/dl or death by stroke or transplantation of two kidneys in marginal conditions). These donor's kidneys present an additional risk of graft loss (13), a greater risk of acute rejection and delay of graft function (14), which is translated into more than ten definitions in the literature, and in 69% of the studies between 1984 and 2007 was defined as the need for dialysis in the first seven days of transplantation (15).

Most clinical studies on the association of calcineurin inhibitors with mTORi excluded patients with expanded criteria donors (16). Experimental studies suggest that combining a calcineurin inhibitor with mTORi interferes with cell metabolism, limiting recovery from injury by ischemia-reperfusion in grafts with less functional reserve, such as in kidneys with high KDPI (17). The study by Ferreira et al. in 2019 demonstrated worse renal function at the end of 12 months in the mTORi group, which presented an imbalance with a high proportion of donors with creatinine above 1.5 mg/dl (18).

In recent years, the Transplant service at HC UNESP, as well as other Brazilian services, has changed the immunosuppression protocol to tacrolimus associated with mTORi in non-sensitized patients for economic reasons to reduce the costs associated with treatment with cytomegalovirus. It created a natural experiment allowing comparison between tacrolimus associated with mycophenolate-based and mTORi-based regimens.

Considering the scarcity of studies related to the topic, the present study aims to compare the regimen containing tacrolimus associated with mycophenolate (TAC-MMF) with tacrolimus associated with mTORi (TAC-mTORi) on renal function at the end of 12 months in four Brazilian centers, subdividing the sample into ranges of “Kidney Donor Profile Index” (KDPI), a tool that evaluates multiple donor characteristics which allows calculating the profile of a kidney graft (19) and has established itself as the most effective scoring system in the USA in terms of assessing the individual risk of a kidney from a deceased donor (20).

## Methods

This is a cohort study of kidney transplant patients performed at UNESP Kidney Transplant Service and three other Brazilian centers over ten years (2008–2018), comparing two immunosuppression regimens: tacrolimus associated with mycophenolate to tacrolimus associated with mTORi in the use of *de novo* software, to analyze kidney function at the end of the first year after transplantation. Data were collected via the HC UNESP medical records system and collaboratively from the other centers involved (São Paulo, Fortaleza, and Juiz de Fora). The groups were divided into tacrolimus associated with mycophenolate (TAC/MMF) and tacrolimus associated with mTORi (TAC/mTORi), and the propensity score was performed aiming at the uniformity of baseline clinical characteristics between the two groups to obtain a control group (TAC/MMF) as similar as possible to the study group (TAC/mTORi). The sample was stratified into KDPI ranges (below 50, from 50 to 85, and above 85) based on the median to test the hypothesis that a donor with high KDPI has an inferior kidney function when using mTORi. The stratification values were chosen by the researchers aiming for clinical relevance, in which values below 50 reflected kidneys with a better prognosis, from 50 to 85 intermediate kidneys, and above 85 with a worse prognosis.

### Immunosuppression protocol

#### Tacrolimus associated with mycophenolate (TAC/MMF) regimen

The regimen consists of the use of tacrolimus (starting with 0.2 mg/kg/day divided into two doses adjusted according to the serum level, aiming at levels of 8–12 ng/ml in the first month and 4–8 ng/ml afterward), mycophenolate sodium 1,440 mg/day divided into two doses, and prednisone 30 mg/day (in the first month with weekly reductions until reaching 5 mg/day at the end of the third month). Induction consists of Basiliximab 20 mg on the day of transplantation and the fourth day (panel-reactive antibody less than 30%) or Thymoglobulin (human anti-thymocyte immunoglobulin) at a dose of 4.5 mg/kg/day (patients with panel-reactive antibody greater than 30%). Mycophenolate sodium dose reduction up to 720 mg/day may occur due to side effects at clinical criterion during follow-up.

#### Tacrolimus associated with mTORi (TAC/mTORi) regimen

The regimen consists of the use of tacrolimus (starting with 0.1 mg/kg/day divided into two intakes adjusted to levels of 4–8 ng/ml until the third month and 2–5 ng/ml thereafter). For mTORi, the use is sirolimus 2 mg/day (adjusted for serum levels of 4–8 ng/ml) or everolimus 1.5 mg every 12 h (adjusted for levels of 3–8 ng/ml). It is associated with Prednisone 30 mg/day (in the first month with weekly reductions until reaching 5 mg/day at the end of the third month). Induction therapy is performed with Thymoglobulin (human anti-thymocyte immunoglobulin) at a dose of 3 mg/kg/day—a single dose. Some patients over 60 may be induced with Basiliximab at the treating physician's discretion. Contraindications for prescribing tacrolimus associated with mTORi are panel-reactivity greater than 30%, presence of anti-donor antibody, FSGS underlying disease, history of thrombotic microangiopathy, BMI greater than 35, and prioritized patients.

#### Prophylactic measures against cytomegalovirus infections

Kidney transplant recipients with a higher risk of cytomegalovirus replication (thymoglobulin with tacrolimus-mycophenolate use or serological mismatch between the donor and the recipient—the recipient is CMV seronegative, and the donor is seropositive) were monitored using the CMV antigenemia assay weekly for the first two months after transplantation and biweekly for the three months after transplantation.

##### Ethical aspects

The basic ethical principles of the guidelines and regulatory standards for research on human beings—according to Resolution 196/96—were followed in the study in question. This research project was submitted to the Human Research Ethics Committee, assessed, and approved (protocol CAAE-23843519.1.0000.5411). All participants who participated in the research were informed about the present study. Its objectives and expected results, and only participated after information and consent, signing the Free and Informed Consent Form.

### Statistical analysis

#### Sample size

The sample was of convenience, including the entire population of kidney transplants over 18 years old with kidneys from deceased donors in the four Brazilian centers between 2008 and 2018. Kidney transplant recipients from a living donor, patients with other classes of immunosuppressants, and patients with a deceased donor under 18 years old were excluded from the sample.

#### Group analysis

For continuous variables, Student's t-test was used for parametric distribution (comparing the two groups) and the Mann-Whitney test for non-parametric variables (comparing the two groups). For categorical variables, the chi-square test or Fisher's exact test was used when appropriate. The outcomes analyzed were death plus graft loss, estimated renal function at 12 months, and estimated renal function with imputation of zero for cases of loss or death. Aiming for better homogeneity of the sample, the propensity score was used with the two immunosuppression regimens as groups by the logistic regression method. The variables selected for this score were age, sex, skin color, underlying disease, cause of death, mismatches, rejection, presence of cytomegalovirus, and final creatinine at 12 months with eGFR by CKD EPI and KDPI. After calculating the propensity score, the method used to select the groups (“match”) was the K-Nearest neighbor (KNN) method using a 0.2 caliper. Balance analysis was performed through visual analysis of QQ normality graphs. New analyses were performed on this new balanced sample, and two different sub-samples were constituted based on the median KDPI. For these analyses, the R 3.4.2 software and the MatchIt package were used.

## Results

Data from 2008 to 2018 were collected from patients using tacrolimus associated with mycophenolate (TAC/MMF) compared to tacrolimus associated with mTORi (TAC/mTORi) as a service protocol. The total number of cases analyzed was 870 (Supplementary Table S1). The overall distribution of cases by immunosuppressant group was 489 for the mTORi group and 381 for the MMF group. Regarding the distribution by centers, Botucatu had 278 cases (32%), Fortaleza with 155 (18%), Juiz de Fora with 268 (31%), and Sao Paulo with 169 cases (19%).

The mean age was similar in both groups, with 50 in the mTORi group and 53 in the MMF group. Regarding race, 85.6% of patients in each group were classified as “non-black” and 14.4% of each group as “black”, and regarding gender, males were more prevalent in both groups (70% mTORi / 69% MMF). Among the renal therapy modalities, most patients were on hemodialysis in both groups (92.8% mTORi and 94% MMF) with a similar average dialysis time (32 months in the mTORi group and 33 in the MMF group). Regarding the underlying disease in the general assessment, the undetermined cause (UNDET) has the highest number of patients (312-36%), followed by diabetes mellitus (DM) with 202-34%, arterial hypertension (AH) 140-16% and chronic glomerulonephritis (CNG) with 94-11%. Seventeen patients (2.5%) were retransplants, 6 (1.5%) in the mTORi group and 11 (3.7%) in the MMF group. The presence of mismatches was also evaluated, with an average of 3 in both groups.

In evaluating donor data, the overall mean age was 46, 44 in the mTORi group, and 51 in the MMF group. The leading causes of death, both overall and by group, were stroke (477-54.8% overall, 233-47.6% mTORi, and 244-64% MMF) and traumatic brain injury (328-37.7% overall, 217-44.4% mTORi, and 111-29.2% MMF). AH was present in 239 of the donors in the general assessment, with 124 (25%) in the mTORi group and 115 (30%) in the MMF group, and diabetes mellitus was present in 49 donors (5.6%) with 28 (5.7%) in the mTORi group and 21 (5.5%) on the MMF. Mean donor creatinine was similar in both groups (1.2 mg/dl). In the overall evaluation of kidney graft characteristics, 575 (66%) were kidneys with standard features and 295 (34%) with expanded criteria. In the mTORi group, 359 (73%) were standard, and 130 (27%) expanded criteria, while in the MMF group, 216 (57%) and 165 (43%) were standard and expanded, respectively. Mean KDPI was 57 in the overall assessment, 48 in the mTORi group, and 66 in the MMF group. Regarding induction, 688 (79%) of the cases used Thymoglobulin, and regarding delayed graft function, 440 (52%) required dialysis in the first week of transplantation, with 204 (54%) in the MMF group. Cytomegalovirus infection was present in 117 (17%) patients, with the majority in the MMF group (78-26%), *p* < 0.001, and graft rejection occurred in 51 patients (7.3%) with similarity between groups, 25 (6.3%) mTORi and 26 (8.7%) MMF, *p* = 0.2. Mortality and graft loss occurred in 164 patients (19%), 79 (16%) in the mTORi group, and 85 (22%) on MMF, *p* = 0.021.

**Figure 1:**
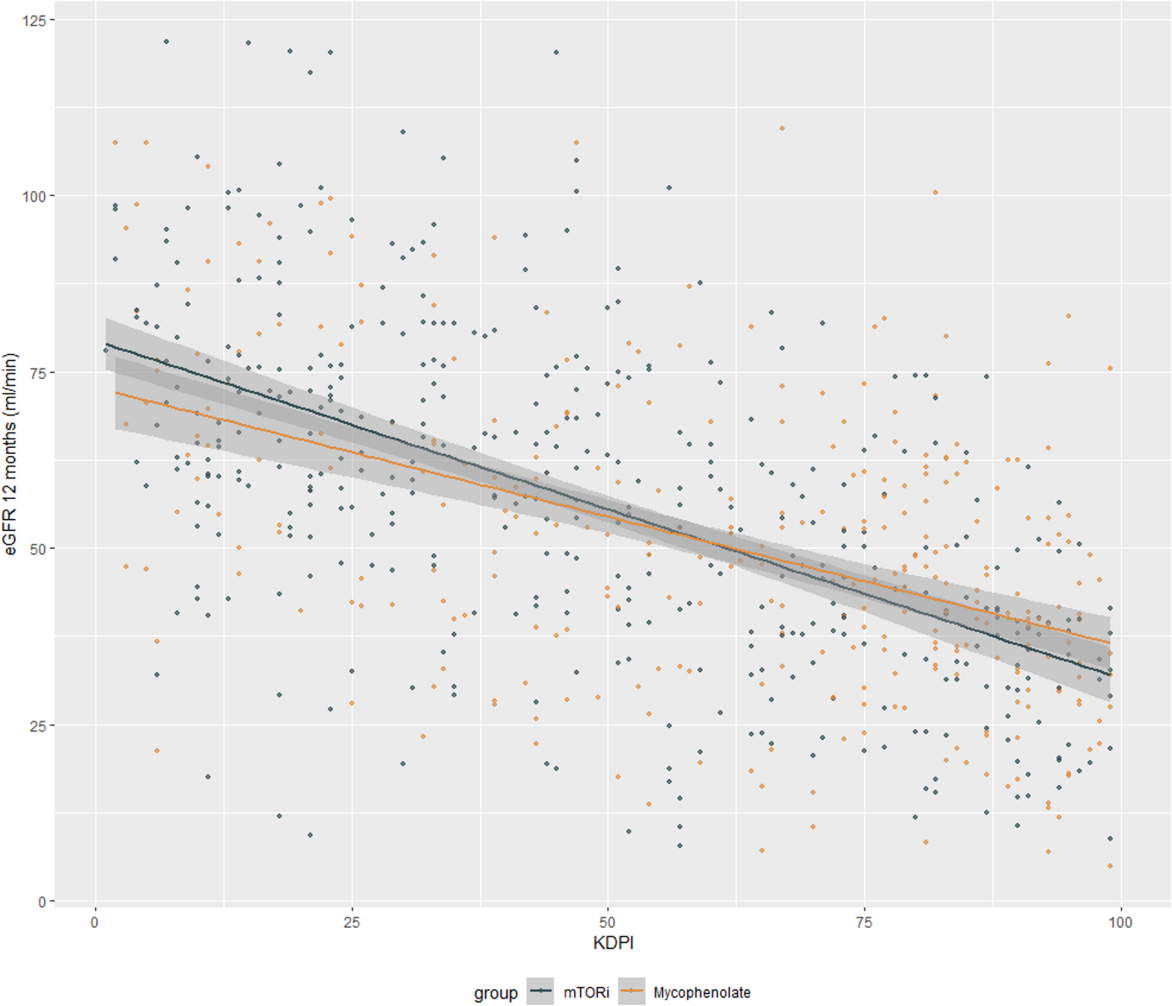
Glomerular filtration rate estimated using the CKD EPI at the end of 12 months of kidney transplantation.

The average glomerular filtration rate estimated using the CKD EPI (Chronic Kidney Epidemiology Collaboration) at the end of 12 months of transplantation was 53 ml/min/m2 with 56 ml/min/m2 in the mTORi group and 48 ml/min/m2 in MMF, *p* < 0.001. When evaluating the average imputed estimated glomerular filtration rate, the mean was 44 ml/min/m2 overall, with 49 ml/min/m2 in the mTORi, and 41 ml/min/m2 in the MMF, *p* < 0.001 (Figure 1).

**Figure 2:**
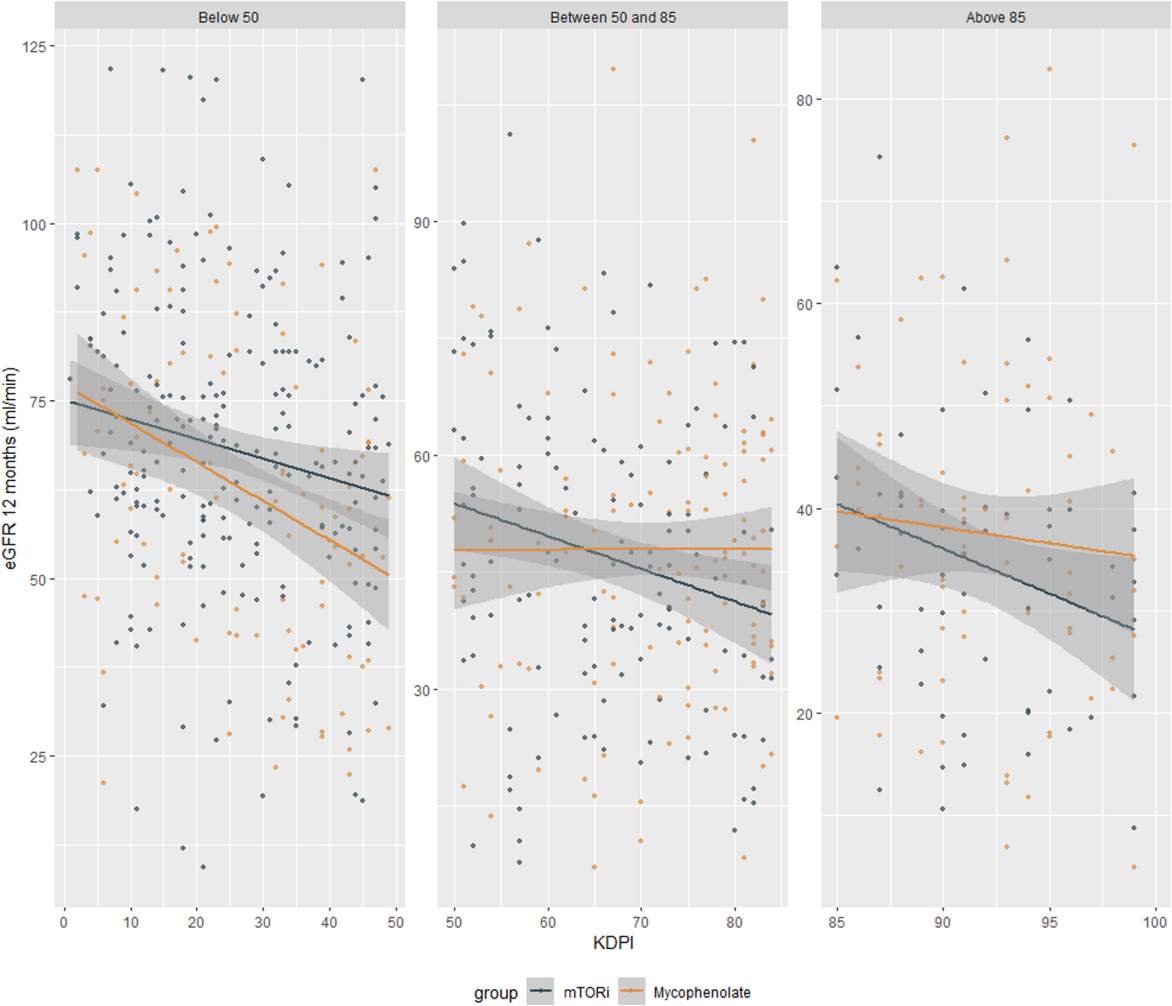
Glomerular filtration rate estimated using the CKD EPI at the end of 12 months of kidney transplantation by KDPI strata.

When evaluating the estimated glomerular filtration rate at the end of 12 months after transplantation separated by KDPI strata, there was no difference between groups. In the stratum of KDPI below 50 (121 patients in each group), the mean eGFR was 64 in the mTORi group and 63 in the MMF group (*p* = 0.4), whereas in the 50–85 stratum (141 patients in each group), the mean eGFR was 46 mTORi and 48 MMF (*p* = 0.4), and in the stratum above 85 (63 patients in each group), it was 36 mTORi and 39 MMF (*p* = 0.2). There was also no difference in the imputed mean, with means of 61 in the mTORi group and 53 in the MMF in the stratum below 50 (*p* = 0.065), means of 40 mTORi and 41 MMF in the stratum from 50 to 85 (*p* = 0.8), and averages of 30 mTORi and 34 MMF in the stratum below 85, *p* = 0.2 (Figure 2).

Regarding the KDPI stratum below 50 (Supplementary Table S2), 119 patients (49%) were from the Botucatu center, 45 (19%) from Fortaleza, and 78 (32%) from Juiz de Fora. The mean age was 52 in the mTORi group and 53 in the MMF group. Males were more prevalent in both groups (87 patients—72% in mTORi and 84-69% in MMF). Race was similar between groups, with the non-black race having 103 (85%) in the mTORi group and 105 (87%) in the MMF, while the black race having 18 (15%) mTORi and 16 (13%) MMF. Among the renal therapy modalities, hemodialysis was the most prevalent in both groups (69 mTORi—89.6%—and 109 MMF—90.9%), and regarding the duration of renal therapy, the averages were 30 months mTORi and 36 months MMF. Regarding the underlying disease, the cause of undetermined origin was the most frequent, with 41 patients (34%) in the mTORi group and 33 (27%) in the MMF group. Three patients (2%) in the mTORi group and 5 (4%) in the MMF group were retransplanted. The mean mismatches were similar, with 3 for each group, and the mean donor creatinine was 1.09 mg/dl in mTORi and 1.0 mg/dl in MMF. 100% (121 in each group) of the transplanted kidneys were standard criteria, and the mean KDPI was 26 in both groups. Cold ischemia time was similar in both groups (16 h mTORi and 12 h MMF). Induction with Thymoglobulin was performed in 113 patients (93%) in the mTORi group and 39 (32%) in the MMF, while induction with Simulect (Basiliximab) was performed in 8 patients (7%) in the mTORi and 82 (68%) MMF. The need for dialysis in the first week of transplantation occurred in 42 (36%) transplanted patients in the mTORi group and 64 (54%) in the MMF group. The rate of cytomegalovirus infection was lower in the mTORi group (10—8%), whereas in the MMF group occurred in 32 patients (26%), *p* < 0.001. There was no difference in the prevalence of rejection with 6 cases (5%) mTORi and 9 (7%) MMF, *p* = 0.4, and when mortality and graft loss were evaluated, they were present in 17 (14%) mTORi and 28 (23%) MMF, *p* = 0.069.

In the KDPI stratum between 50 and 85 (Supplementary Table S3), 105 patients (37.3%) were from Botucatu center, 16 (5.7%) from Fortaleza, 102 (36.1%) from Juiz de Fora, and 59 (20.9%) from Sao Paulo. The mean age was 54 in both groups. Race was similar between groups, with the non-black race having 120 (85%) in both groups and the black race having 21 (15%) in both groups. The male gender prevailed in both groups, with 102 patients—72% in mTORi and 96%–68% in MMF.

Regarding the renal therapy modalities, hemodialysis was the most prevalent in both groups (121 mTORi—97%—and 133 MMF—95%), and regarding the duration of renal therapy, the averages were 30 months mTORi and 29 months MMF. Regarding the underlying disease, the cause of undetermined origin was the most frequent, with 48 patients (34%) in the mTORi group and 43 (30%) in the MMF group. Two (2%) and 3 (3%) patients were retransplanted. The mean mismatches were similar at 3 for each group, and the mean donor creatinine was 1.3 mg/dl in both groups. In the evaluation of renal graft characteristics, 98 (70%) in the mTORi group and 75 (53%) in the MMF group were standard kidneys, whereas 43 (30%) mTORi and 66 (47%) MMF were expanded criteria. The mean KDPI was 68 mTORi and 73 MMF. Cold ischemia time was similar in both groups (17 h mTORi and 15 h MMF). Induction with Thymoglobulin was performed in 131 patients (93%) in the mTORi group and 83 (59%) in the MMF group, while induction with Simulect (Basiliximab) was performed in 10 patients (7%) in the mTORi group and 58 (41%) in the MMF group. The need for dialysis in the first week of transplantation occurred in 73 (53%) transplanted patients in both groups. The rate of cytomegalovirus infection was lower in the mTORi group (8-7%), whereas in the MMF group, it occurred in 31 patients (30%), *p* < 0.001. There was no difference in the prevalence of rejection, with 9 cases (8%) in the mTORi group and 10 cases (10%) in the MMF group, *p* = 0.7. The occurrence of death and graft loss happened in 28 (20%) in mTORi and 31 (22%) in MMF, *p* = 0.7.

In the KDPI stratum above 85 (Supplementary Table S4), 18 patients (14.3%) were from Botucatu, 22 (17.5%) from Fortaleza and 86 (68.2%) from Sao Paulo. The mean age was 52 in the mTORi group and 56 in the MMF group. Race was similar between groups, with the non-black race having 48 (76%) in the mTORi group and 54 (86%) in the MMF, while the black race, with 15 (24%) mTORi and 9 (14%) MMF. The male gender prevailed in both groups (46 patients in mTORi—73%—and 48 in MMF—76%). Among the renal therapy modalities, hemodialysis was the most prevalent in both groups (60 mTORi—95%—and 59 MMF—94%), and regarding the duration of renal therapy, the averages were 32 months mTORi and 34 months MMF. Regarding the underlying disease, the cause of undetermined origin was the most frequent, with 28 patients (44%) in the mTORi group and 32 (51%) in the MMF group. Regarding retransplantation, there were no patients. The mean mismatches were 2 for the mTORi group and 3 for the MMF, and the mean donor creatinine was 1.4 mg/dl in both groups. In the evaluation of renal graft characteristics, none in the mTORi group and 1 (2%) in the MMF group was a standard kidney, whereas 63 (100%) mTORi and 62 (98%) MMF were expanded criteria. Mean KDPI was 92 mTORi and 91 MMF. Cold ischemia time was similar in both groups (20.7 h mTORi and 21 h MMF), and induction with Thymoglobulin was performed in 62 patients (98%) in the mTORi group and 53 (84%) in the MMF group, whereas induction with Simulect (Basiliximab), was performed in 1 patient (2%) on mTORi and 10 (16%) on MMF. The need for dialysis in the first week of transplantation occurred in 35 (56%) transplanted patients in the mTORi group and 41 (65%) in the MMF group. The cytomegalovirus infection rate was zero in the mTORi group, while in the MMF group, it occurred in 9 patients (41%), *p* = 0.002. There was no graft rejection in the mTORi group, and in the MMF group, it happened in 3 cases (14%), *p* = 0.2. Regarding deaths and graft loss, they occurred in 16 (25%) in mTORi and 13 (21%) in MMF, *p* = 0.5.

## Discussion

The current standard immunosuppressive regimen consists of tacrolimus, mycophenolate, and steroids, which, despite excellent short-term outcomes, can have relevant side effects and poor long-term outcomes (21). An alternative studied in recent years is the association of tacrolimus with mTORi. However, there is a lack of studies proving the effectiveness of this regimen in patients with high KDPI. The present study evaluated patients from four Brazilian centers and stratified by KDPI ranges (below 50, from 50 to 85, and above 85).

The studied centers (Botucatu, Fortaleza, Juiz de Fora, and Sao Paulo) presented data with different profiles, reflecting the characteristics of the kidneys offered in each region. In the case of our study, the transplant center in the capital of Sao Paulo did not have any participants in the KDPI stratum below 50, while the center in Fortaleza did not have any participants in the stratum above 85.

The unmatched global analysis showed renal function at the end of 12 months (both imputed and non-imputed) better in the mTORi group. However, this group had lower KDPI and younger recipient age, factors related to better outcomes. When evaluating the match stratification, all KDPI strata showed no differences between the mTORi and mycophenolate groups. Most large studies related to the comparison of the two immunosuppressive regimens do not directly address KDPI but bring similar efficacy and kidney function compared between groups. The A2309 Study from 2010 (22) evaluated 833 patients and demonstrated non-inferiority of the mTORi group (everolimus 1.5 and 3 mg) vs. mycophenolate with eGFR 54.6 and 51.3 vs. 52.2 ml/min/1.73 m2 respectively. As well as the TRANSFORM study (7), which evaluated over 2,000 patients, also demonstrated non-inferiority at the end of 12 months [48.2% (493 of 1,022 patients) mTORi vs. 45.1% (457 of 1,015) mycophenolate, a difference of 3. 2% with 95% CI: 1.3%–7.6%] and later at the end of 24 months. The US92 north-american study (23), which evaluated 613 patients, was also able to show comparable safety with the two regimens [48.2% (493) in the mTORi group and 45.1% (457) in the mycophenolate group (3.2% difference; 95% CI: 1.3%–7.6%).

Regarding ATHENA (24), a European multicenter study with 655 participants, the result showed worse renal function at the end of one year in the mTORi group (62.2 ml/min/1.73 m2 in the everolimus/tacrolimus group, 58.4 ml/min/m2 in the everolimus/cyclosporine group, and 67.8 ml/min/m2 in the mycophenolate group, *p* = 0.007 and *p* < 0.001). In a *post hoc* analysis, the subgroup of patients who used tacrolimus and had levels below or equal to 5 ng/ml, renal function at the end of 12 months did not differ between groups (64.9 ml/min/1.73 m2 mTORi vs. 70.3 ml/min/1.73 m2 mycophenolate). This difference in the initial assessment was explained by the fact that the regimen used a standard dose of the calcineurin inhibitor, unlike previous studies and the *post hoc* analysis that used low doses of tacrolimus, thus showing that the dose may influence the results given the nephrotoxic potential of this drug class.

When evaluating deaths and graft loss between groups in strata, we found no difference. Montero et al., in their 2019 meta-analysis that evaluated a total of 24 studies with 7,356 patients, presented 3.468 cases of graft loss in the mTORi group and 2.783 in the mycophenolate group [RR 1.02 (0.74–1.40)] at the end of the first year and 1,510 in the mTORi group and 850 mycophenolate [RR 1.25 (0.95–1.66)] at 48 months (25). The study by Xie et al. in 2015 evaluated 11 studies with 4,630 patients [RR 1.2 (1.02–1.4) *p* = 0.03] (26) carrying a higher risk of graft loss associated with the mTORi group. However, after performing a better analysis, such a pattern was seen in patients who used a standard dose of CI, reinforcing the hypothesis of immunological synergy. With the better design of the studies over the last years, the data bring higher agreement with this work. In the evaluation of deaths, Montero et al. also found no difference (RR 1.01 - 0.74, 1.38), as well as the ATHENA multicenter study (4.8%, 6.5% and 2.9% in the everolimus-EVR/tacrolimus-TAC, everolimus/cyclosporine, and mycophenolate/tacrolimus groups, respectively—*p* = 0.294 for EVR/ TAC vs. mycophenolate/TAC and *p* = 0.082 for EVR/cyclosporine and mycophenolate/TAC) (24).

In the rejection assessment, there were no differences across all KDPI strata. Studies such as the one by HUH et al. in 2017 with 159 patients brought a greater favorability in the rejection rate in the mTORi group. However, without statistical significance (5.3% in the mTORi group and 13.3 in the mycophenolate group *p* = 0, 09) (27).

As shown in the ATHENA study (24) and the study by Qazi et al. in 2,017 (27), there are differences in viral infection between mTORi and mycophenolate with a possible protective mechanism of mTORi (ATHENA—6.2% and 2.5% in groups with mTORi and 20.6% in the mycophenolate group with *p* < 0.01; Qazi et al.—4.2% mTORi and 8.2% mycophenolate with *p* < 0.05). Our study also found differences showing a lower incidence of cytomegalovirus across all KDPI strata in the mTORi group. The inhibition of viral protein translation and stimulation of specific T cells against viruses may be biological factors that help to understand this effect (28).

The study has strengths such as the participation of four major Brazilian reference centers in kidney transplantation, as well as the use of data from transplants performed over a wide period (ten years). The study also presents some limitations, such as failure to collect some data, being classified as unknown, or with grouped results like in the case of death or graft loss. Other limitations were the failure to evaluate the serial serum dosage of immunosuppressants to correlate with the outcomes and the use of KDPI with wide margins, which may have hidden some differences that could appear in the case of strata with lower margins.

In conclusion, this study showed that the regimen using mTORi inhibitor is an effective and safe regimen when compared to the standard regimen. In addition, the scheme seems to offer additional protection against cytomegalovirus infections and may be a relevant ally in cases of high risk for these pathologies.

## Data Availability

The original contributions presented in the study are included in the article/**Supplementary Material**, further inquiries can be directed to the corresponding author.
