# Supplementary material for: Renal function at 12 months of kidney transplantation comparing tacrolimus and mycophenolate with tacrolimus and mTORi in donors with different KDPI ranges. A multicenter cohort study using propensity scoring
Source: Front Transplant. 2023 Oct 16;2:1279940. doi: 10.3389/frtra.2023.1279940 (PMC11235318; doi:10.3389/frtra.2023.1279940)
Supplement: Supplementary file 1 [file Table1.docx]

Supplementary Material

# Supplementary Tables

Table 1 - Clinical and laboratory characteristics of kidney transplant patients

| **Variables** | **Overall** N = 870 | **mTORi** N = 489 | **MMF** N = 381 | **p-value** |
| --- | --- | --- | --- | --- |
| **Center** |  |  |  | <0.001 |
| Botucatu | 278 (32%) | 142 (29%) | 136 (36%) |  |
| Fortaleza | 155 (18%) | 153 (31%) | 2 (0.5%) |  |
| Juiz de Fora | 268 (31%) | 106 (22%) | 162 (42.5%) |  |
| São Paulo | 169 (19%) | 88 (18%) | 81 (21%) |  |
| **Age** | 52 (41, 60) | 50 (38, 60) | 53 (45, 59) | 0.002 |
| **Race** |  |  |  | >0.9 |
| Non-black | 744 (86%) | 418 (86%) | 326 (86%) |  |
| Black | 125 (14%) | 70 (14%) | 55 (14%) |  |
| Unknown | 1 | 1 | 0 |  |
| **Gender** |  |  |  | 0.7 |
| Female | 266 (31%) | 147 (30%) | 119 (31%) |  |
| Male | 604 (69%) | 342 (70%) | 262 (69%) |  |
| **Type of treatment** |  |  |  | 0.6 |
| Hemodialysis | 666 (93.4%) | 310 (92.8%) | 356 (94%) |  |
| Peritoneal | 39 (5.5%) | 21 (6.3%) | 18 (4.7%) |  |
| Preemptive | 8 (1.1%) | 3 (0.9%) | 5 (1.3%) |  |
| Unknown | 157 | 155 | 2 |  |
| **Time on dialysis** | 33 (20, 53) | 32 (20, 54) | 33 (22, 50) | >0.9 |
| Unknown | 277 | 112 | 165 |  |
| **Underlying disease** |  |  |  | 0.015 |
| Diabetes | 202 (23%) | 116 (24%) | 86 (23%) |  |
| Glomerulonephritis | 94 (11%) | 56 (11%) | 38 (10%) |  |
| Hypertension | 140 (16%) | 60 (12%) | 80 (21%) |  |
| Undetermined | 312 (36%) | 186 (38%) | 126 (33%) |  |
| Other | 122 (14%) | 71 (15%) | 51 (13%) |  |
| **Retransplant** |  |  |  | 0.065 |
| No | 684 (97.5%) | 395 (98.5%) | 289 (96.3%) |  |
| Yes | 17 (2.5%) | 6 (1.5%) | 11 (3.7%) |  |
| Unknown | 169 | 88 | 81 |  |
| **Mismatch** | 3.00 (2.00, 4.00) | 3.00 (2.00, 4.00) | 3.00 (2.00, 4.00) | 0.079 |
| Unknown | 3 | 3 | 0 |  |
| **Donor age** | 46 (33, 57) | 44 (31, 55) | 51 (38, 60) | <0.001 |
| **Donor’s detail of death** |  |  |  | <0.001 |
| Stroke | 477 (54.8%) | 233 (47.6%) | 244 (64%) |  |
| Traumatic Brain Injury | 328 (37.7%) | 217 (44.4%) | 111 (29.2%) |  |
| Others | 65 (7.5%) | 39 (8%) | 26 (6.8%) |  |
| **Donor hypertension** |  |  |  | 0.11 |
| No | 631 (73%) | 365 (75%) | 266 (70%) |  |
| Yes | 239 (27%) | 124 (25%) | 115 (30%) |  |
| **Donor diabetes** |  |  |  | 0.5 |
| Unknown | 169 (19.4%) | 88 (18%) | 81 (21.3%) |  |
| No | 652 (75%) | 373 (76.3%) | 279 (73.2%) |  |
| Yes | 49 (5.6%) | 28 (5.7%) | 21 (5.5%) |  |
| **Donor creatinine (mg/dL)** | 1.20 (0.90, 1.80) | 1.20 (0.90, 1.84) | 1.30 (0.90, 1.80) | 0.8 |
| **Criteria** |  |  |  | <0.001 |
| Expanded | 295 (34%) | 130 (27%) | 165 (43%) |  |
| Standard | 575 (66%) | 359 (73%) | 216 (57%) |  |
| **KDPI** | 57 (29, 81) | 48 (24, 76) | 66 (39, 84) | <0.001 |
| **Cold Ischemia Time** | 18 (13, 22) | 19 (14, 23) | 15 (12, 20) | <0.001 |
| Unknown | 6 | 5 | 1 |  |
| **Induction** |  |  |  | <0.001 |
| Simulect | 182 (21%) | 21 (4.3%) | 161 (42%) |  |
| Timo | 688 (79%) | 468 (95.7%) | 220 (58%) |  |
| **Delayed graft function** |  |  |  | 0.14 |
| No | 414 (48%) | 243 (51%) | 171 (46%) |  |
| Yes | 440 (52%) | 236 (49%) | 204 (54%) |  |
| Unknown | 16 | 10 | 6 |  |
| **Cytomegalovirus** |  |  |  | <0.001 |
| No | 578 (83%) | 358 (90.2%) | 220 (74%) |  |
| Yes | 117 (17%) | 39 (9.8%) | 78 (26%) |  |
| Unknown | 175 | 92 | 83 |  |
| **Rejection** |  |  |  | 0.2 |
| No | 644 (92.7%) | 372 (93.7%) | 272 (91.3%) |  |
| Yes | 51 (7.3%) | 25 (6.3%) | 26 (8.7%) |  |
| Unknown | 175 | 92 | 83 |  |
| **Death or graft loss** |  |  |  | 0.021 |
| No | 706 (81%) | 410 (84%) | 296 (78%) |  |
| Yes | 164 (19%) | 79 (16%) | 85 (22%) |  |
| **12-month eGFR** | 53 (38, 69) | 56 (39, 73) | 48 (35, 63) | <0.001 |
| Unknown | 164 | 79 | 85 |  |
| **Imputed 12-month eGFR** | 44 (21, 64) | 49 (25, 69) | 41 (16, 60) | <0.001 |
|  |  |  |  |  |
|  |  |  |  |  |

Table 2 - Clinical and laboratory characteristics of kidney transplant patients in the KDPI stratum below 50

| **Variables** | **Overall** N = 242 | **mTORi** N = 121 | **MMF** N = 121 | **p-value** |
| --- | --- | --- | --- | --- |
| **Center** |  |  |  | <0.001 |
| Botucatu | 119 (49%) | 50 (41.3%) | 69 (57%) |  |
| Fortaleza | 45 (19%) | 44 (36.4%) | 1 (0.8%) |  |
| Juiz de Fora | 78 (32%) | 27 (22.3%) | 51 (42.2%) |  |
| São Paulo | 0 (0%) | 0(0%) | 0 (0%) |  |
| **Age** | 52 (43, 60) | 52 (41, 61) | 53 (45, 59) | 0.9 |
| **Race** |  |  |  | 0.7 |
| Non-black | 208 (86%) | 103 (85%) | 105 (87%) |  |
| Black | 34 (14%) | 18 (15%) | 16 (13%) |  |
| **Gender** |  |  |  | 0.7 |
| Female | 71 (29%) | 34 (28%) | 37 (31%) |  |
| Male | 171 (71%) | 87 (72%) | 84 (69%) |  |
| **Type of treatment** |  |  |  | 0.5 |
| Hemodialysis | 178 (90.3%) | 69 (89.6%) | 109 (90.9%) |  |
| Peritoneal | 14 (7.1%) | 7 (9.1%) | 7 (5.8%) |  |
| Preemptive | 5 (2.6%) | 1 (1.3%) | 4 (3.3%) |  |
| Unknown | 45 | 44 | 1 |  |
| **Time on dialysis** | 31 (19, 53) | 30 (19, 54) | 36 (21, 53) | 0.4 |
| **Underlying disease** |  |  |  | 0.5 |
| Diabetes | 60 (24.8%) | 28 (23%) | 32 (26%) |  |
| Glomerulonephritis | 26 (10.7%) | 13 (10%) | 13 (11%) |  |
| Hypertension | 49 (20.3%) | 20 (17%) | 29 (24%) |  |
| Undetermined | 74 (30.6%) | 41 (34%) | 33 (27%) |  |
| Other | 33 (13.6%) | 19 (16%) | 14 (12%) |  |
| **Retransplant** |  |  |  | 0.7 |
| No | 234 (97%) | 118 (98%) | 116 (96%) |  |
| Yes | 8 (3%) | 3 (2%) | 5 (4%) |  |
| **Mismatch** | 3.00 (3.00, 4.00) | 3.00 (3.00, 4.00) | 3.00 (3.00, 4.00) | 0.4 |
| **Donor age** | 32 (26, 39) | 32 (26, 39) | 32 (25, 40) | >0.9 |
| **Donor’s detail of death** |  |  |  | 0.3 |
| Stroke | 73 (30.2%) | 31 (26%) | 42 (35%) |  |
| Traumatic Brain Injury | 147 (60.7%) | 78 (64%) | 69 (57%) |  |
| Others | 22 (9.1%) | 12 (10%) | 10 (8%) |  |
| **Donor hypertension** |  |  |  | 0.4 |
| No | 229 (95%) | 116 (96%) | 113 (93%) |  |
| Yes | 13 (5%) | 5 (4%) | 8 (7%) |  |
| **Donor diabetes** |  |  |  | 0.4 |
| No | 235 (97%) | 116 (96%) | 119 (98%) |  |
| Yes | 7 (3.0%) | 5 (4%) | 2 (2%) |  |
| **Donor creatinine**  **(mg/dL)** | 1.00 (0.80, 1.50) | 1.09 (0.80, 1.40) | 1.00 (0.80, 1.69) | 0.4 |
| **Criteria** |  |  |  |  |
| Expanded | 0 (0%) | 0 (0%) | 0 (0%) |  |
| Standard | 242 (100%) | 121 (100%) | 121 (100%) |  |
| **KDPI** | 26 (14, 38) | 26 (14, 34) | 26 (13, 39) | 0.7 |
| **Cold Ischemia Time** | 15 (11, 18) | 16 (12, 20) | 14 (11, 18) | 0.11 |
| **Induction** |  |  |  | <0.001 |
| Simulect | 90 (37%) | 8 (7%) | 82 (68%) |  |
| Timo | 152 (63%) | 113 (93%) | 39 (32%) |  |
| **Delayed graft function** |  |  |  | 0.006 |
| No | 130 (55%) | 75 (64%) | 55 (46%) |  |
| Yes | 106 (45%) | 42 (36%) | 64 (54%) |  |
| Unknown | 6 | 4 | 2 |  |
| **Cytomegalovirus** |  |  |  | <0.001 |
| No | 199 (83%) | 110 (92%) | 89 (74%) |  |
| Yes | 42 (17%) | 10 (8%) | 32 (26%) |  |
| Unknown | 1 | 1 | 0 |  |
| **Rejection** |  |  |  | 0.4 |
| No | 226 (93.7%) | 114 (95%) | 112 (93%) |  |
| Yes | 15 (6.3%) | 6 (5%) | 9 (7%) |  |
| Unknown | 1 | 1 | 0 |  |
| **Death or graft loss** |  |  |  | 0.069 |
| No | 197 (81%) | 104 (86%) | 93 (77%) |  |
| Yes | 45 (19%) | 17 (14%) | 28 (23%) |  |
| **12-month eGFR** | 63 (52, 78) | 64 (56, 78) | 63 (47, 79) | 0.4 |
| Unknown | 45 | 17 | 28 |  |
| **Imputed 12-month eGFR** | 60 (31, 76) | 61 (44, 76) | 53 (26, 73) | 0.065 |

Table 3 - Clinical and laboratory characteristics of kidney transplant patients in the KDPI stratum between 50 and 85

| **Variables** | **Overall** N = 282 | **mTORi** N = 141 | **MMF** N = 141 | **p-value** |
| --- | --- | --- | --- | --- |
| **Center** |  |  |  | 0.001 |
| Botucatu | 105 (37.3%) | 56 (39.7%) | 49 (34.7%) |  |
| Fortaleza | 16 (5.7%) | 15 (10.6%) | 1 (0.7%) |  |
| Juiz de Fora | 102 (36.1%) | 46 (32.6%) | 56 (39.7%) |  |
| São Paulo | 59 (20.9%) | 24 (17.1%) | 35 (24.9%) |  |
| **Age** | 54 (43, 62) | 54 (42, 63) | 54 (45, 60) | 0.8 |
| **Race** |  |  |  | >0.9 |
| Non-black | 240 (85%) | 120 (85%) | 120 (85%) |  |
| Black | 42 (15%) | 21 (15%) | 21 (15%) |  |
| **Gender** |  |  |  | 0.4 |
| Female | 84 (30%) | 39 (28%) | 45 (32%) |  |
| Male | 198 (70%) | 102 (72%) | 96 (68%) |  |
| **Type of treatment** |  |  |  | 0.9 |
| Hemodialysis | 254 (95.8%) | 121 (97%) | 133 (95%) |  |
| Peritoneal | 10 (3.8%) | 4 (3%) | 6 (4.3%) |  |
| Preemptive | 1 (0.4%) | 0 (0%) | 1 (0.7%) |  |
| Unknown | 17 | 16 | 1 |  |
| **Time on dialysis** | 30 (19, 46) | 30 (19, 48) | 29 (19, 42) | 0.6 |
| **Underlying disease** |  |  |  | 0.2 |
| Diabetes | 77 (27.3%) | 41 (29%) | 36 (26%) |  |
| Glomerulonephritis | 26 (9.2%) | 14 (10%) | 12 (8%) |  |
| Hypertension | 48 (17.1%) | 16 (11%) | 32 (23%) |  |
| Undetermined | 91 (32.2%) | 48 (34%) | 43 (30%) |  |
| Other | 40 (14.2%) | 22 (16%) | 18 (13%) |  |
| **Retransplant** |  |  |  | 0.7 |
| No | 218 (98%) | 115 (98%) | 103 (97%) |  |
| Yes | 5 (2%) | 2 (2%) | 3 (3%) |  |
| Unknown | 59 | 24 | 35 |  |
| **Mismatch** | 3.00 (2.00, 4.00) | 3.00 (2.00, 4.00) | 3.00 (2.00, 4.00) | 0.8 |
| **Donor age** | 52 (47, 56) | 52 (47, 56) | 52 (47, 57) | 0.6 |
| **Donor’s detail of death** |  |  |  | 0.3 |
| Stroke | 210 (74.4%) | 100 (71%) | 110 (78%) |  |
| Traumatic Brain Injury | 52 (18.5%) | 31 (22%) | 21 (15%) |  |
| Others | 20 (7.1%) | 10 (7%) | 10 (7%) |  |
| **Donor hypertension** |  |  |  | 0.4 |
| No | 175 (62%) | 91 (65%) | 84 (60%) |  |
| Yes | 107 (38%) | 50 (35%) | 57 (40%) |  |
| **Donor diabetes** |  |  |  | 0.2 |
| No | 205 (73%) | 106 (75%) | 99 (70%) |  |
| Yes | 18 (6%) | 11 (8%) | 7 (5%) |  |
| Unknown | 59 (21%) | 24 (17%) | 35 (25%) |  |
| **Donor creatinine**  **(mg/dL)** | 1.30 (0.94, 1.80) | 1.30 (0.93, 1.80) | 1.30 (0.98, 1.80) | 0.8 |
| **Criteria** |  |  |  | 0.005 |
| Expanded | 109 (39%) | 43 (30%) | 66 (47%) |  |
| Standard | 173 (61%) | 98 (70%) | 75 (53%) |  |
| **KDPI** | 70 (60, 78) | 68 (60, 77) | 73 (62, 79) | 0.042 |
| **Cold Ischemia Time** | 16 (12, 20) | 17 (12, 21) | 15 (12, 20) | 0.4 |
| **Induction** |  |  |  | <0.001 |
| Simulect | 68 (24%) | 10 (7%) | 58 (41%) |  |
| Timo | 214 (76%) | 131 (93%) | 83 (59%) |  |
| **Delayed graft function** |  |  |  | >0.9 |
| No | 128 (47%) | 64 (47%) | 64 (47%) |  |
| Yes | 146 (53%) | 73 (53%) | 73 (53%) |  |
| Unknown | 8 | 4 | 4 |  |
| **Cytomegalovirus** |  |  |  | <0.001 |
| No | 180 (82%) | 107 (93%) | 73 (70%) |  |
| Yes | 39 (18%) | 8 (7%) | 31 (30%) |  |
| Unknown | 63 | 26 | 37 |  |
| **Rejection** |  |  |  | 0.7 |
| No | 199 (91%) | 105 (92%) | 94 (90%) |  |
| Yes | 19 (9%) | 9 (8%) | 10 (10%) |  |
| Unknown | 64 | 27 | 37 |  |
| **Death or graft loss** |  |  |  | 0.7 |
| No | 223 (79%) | 113 (80%) | 110 (78%) |  |
| Yes | 59 (21%) | 28 (20%) | 31 (22%) |  |
| **12-month eGFR** | 46 (33, 59) | 46 (33, 58) | 48 (35, 60) | 0.4 |
| Unknown | 59 | 28 | 31 |  |
| **Imputed 12-month eGFR** | 41 (16, 54) | 40 (17, 54) | 41 (16, 55) | 0.8 |

Table 4 - Clinical and laboratory characteristics of kidney transplant patients in the KDPI stratum above 85

| **Variables** | **Overall** N = 126 | **mTORi** N = 63 | **MMF** N = 63 | **p-value** |
| --- | --- | --- | --- | --- |
| **Center** |  |  |  | 0.7 |
| Botucatu | 18 (14.3%) | 8 (13%) | 10 (16%) |  |
| Juiz de Fora | 22 (17.5%) | 10 (16%) | 12 (19%) |  |
| São Paulo  Fortaleza | 86 (68.2%)  0 (0%) | 45 (71%)  0 (0%) | 41 (65%)  0 (0%) |  |
| **Age** | 53 (44, 61) | 52 (41, 61) | 56 (46, 60) | 0.6 |
| **Race** |  |  |  | 0.2 |
| Non-black | 102 (81%) | 48 (76%) | 54 (86%) |  |
| Black | 24 (19%) | 15 (24%) | 9 (14%) |  |
| **Gender** |  |  |  | 0.7 |
| Female | 32 (25%) | 17 (27%) | 15 (24%) |  |
| Male | 94 (75%) | 46 (73%) | 48 (76%) |  |
| **Type of treatment** |  |  |  | >0.9 |
| Hemodialysis | 119 (94%) | 60 (95%) | 59 (94%) |  |
| Peritoneal | 7 (6%) | 3 (5%) | 4 (6%) |  |
| **Time on dialysis** | 34 (22, 49) | 32 (20, 48) | 34 (24, 50) | 0.6 |
| **Underlying disease** |  |  |  | 0.4 |
| Diabetes | 26 (21%) | 17 (27%) | 9 (14%) |  |
| Glomerulonephritis | 14 (11%) | 8 (13%) | 6 (10%) |  |
| Hypertension | 13 (10%) | 5 (8%) | 8 (12%) |  |
| Undetermined | 60 (48%) | 28 (44%) | 32 (51%) |  |
| Other | 13 (10%) | 5 (8%) | 8 (13%) |  |
| **Retransplant** |  |  |  |  |
| No | 40 (100%) | 18 (100%) | 22 (100%) |  |
| Unknown | 86 | 45 | 41 |  |
| **Mismatch** | 3.00 (2.00, 3.00) | 2.00 (2.00, 3.00) | 3.00 (2.00, 3.00) | 0.4 |
| **Donor age** | 64.0 (60.0, 67.0) | 64.0 (60.0, 67.5) | 64.0 (60.0, 67.0) | 0.8 |
| **Donor’s detail of death** |  |  |  | 0.9 |
| Stroke | 103 (82%) | 50 (79%) | 53 (84%) |  |
| Traumatic Brain Injury | 17 (13%) | 10 (16%) | 7 (11%) |  |
| Others | 6 (5%) | 3 (5%) | 3 (5%) |  |
| **Donor hypertension** |  |  |  | >0.9 |
| No | 42 (33%) | 21 (33%) | 21 (33%) |  |
| Yes | 84 (67%) | 42 (67%) | 42 (67%) |  |
| **Donor diabetes** |  |  |  | 0.7 |
| No | 30 (24%) | 13 (21%) | 17 (27%) |  |
| Yes | 10 (8%) | 5 (8%) | 5 (8%) |  |
| Unknown | 86 (68%) | 45 (71%) | 41 (65%) |  |
| **Donor creatinine**  **(mg/dL)** | 1.40 (1.00, 2.48) | 1.40 (0.93, 2.60) | 1.40 (1.15, 2.30) | 0.5 |
| **Criteria** |  |  |  | >0.9 |
| Expanded | 125 (99%) | 63 (100%) | 62 (98%) |  |
| Standard | 1 (1%) | 0 (0%) | 1 (2%) |  |
| **KDPI** | 91.5 (88.0, 95.0) | 92.0 (88.0, 95.0) | 91.0 (89.0, 95.0) | 0.9 |
| **Cold Ischemia Time** | 20.8 (17.0, 24.7) | 20.7 (17.0, 24.0) | 21.0 (16.3, 25.2) | >0.9 |
| **Induction** |  |  |  | 0.005 |
| Simulect | 11 (9%) | 1 (2%) | 10 (16%) |  |
| Timo | 115 (91%) | 62 (98%) | 53 (84%) |  |
| **Delayed graft function** |  |  |  | 0.3 |
| No | 49 (39%) | 27 (44%) | 22 (35%) |  |
| Yes | 76 (61%) | 35 (56%) | 41 (65%) |  |
| Unknown | 1 | 1 | 0 |  |
| **Cytomegalovirus** |  |  |  | 0.002 |
| No | 31 (78%) | 18 (100%) | 13 (59%) |  |
| Yes | 9 (22%) | 0 (0%) | 9 (41%) |  |
| Unknown | 86 | 45 | 41 |  |
| **Rejection** |  |  |  | 0.2 |
| No | 37 (92%) | 18 (100%) | 19 (86%) |  |
| Yes | 3 (8%) | 0 (0%) | 3 (14%) |  |
| Unknown | 86 | 45 | 41 |  |
| **Death or graft loss** |  |  |  | 0.5 |
| No | 97 (77%) | 47 (75%) | 50 (79%) |  |
| Yes | 29 (23%) | 16 (25%) | 13 (21%) |  |
| **12-month eGFR** | 38 (28, 45) | 36 (24, 41) | 39 (30, 50) | 0.2 |
| Unknown | 29 | 16 | 13 |  |
| **Imputed 12-month eGFR** | 32 (11, 41) | 30 (4, 40) | 34 (16, 43) | 0.2 |
