# Supplementary material for: Renal function at 12 months of kidney transplantation comparing tacrolimus and mycophenolate with tacrolimus and mTORi in donors with different KDPI ranges. A multicenter cohort study using propensity scoring
Source: Front Transplant. 2023 Oct 16;2:1279940. doi: 10.3389/frtra.2023.1279940 (PMC11235318; doi:10.3389/frtra.2023.1279940)
Supplement: Supplementary file 2 [file Datasheet1.pdf]

Supplementary Material

1 Supplementary Data

Appendix A

UNESP -FACULDADE DE MEDICINA DE BOTUCATU

PARECER CONSUBSTANCIADO DO CEP

DADOS DO PROJETO DE PESQUISA

Título da Pesquisa: Função renal durante 12 meses de transplante comparando tacrolimo e micofenolato com
tacrolimo e imTOR em doadores com diferentes faixas de KDPI. Um estudo retrospectivo
utilizando escore de propensão.

Pesquisador: ARLISSON MACEDO RODRIGUES

Área Temática:

Versão: 3

CAAE: 23843519.1.0000.5411

Instituição Proponente: Departamento de Clínica Médica

Patrocinador Principal: Financiamento Próprio

DADOS DO PARECER

Número do Parecer: 3.798.291

Apresentação do Projeto:

As informações descritas nos campos "Apresentação do Projeto", "Objetivo da Pesquisa" e "Avaliação dos Riscos e Benefícios" foram retiradas dos documentos e arquivo - Informações Básicas da Pesquisa.
A doença renal crônica (DRC) é uma condição relacionada a um risco aumentado de internação hospitalar, morbidade e mortalidade por doença cardiovascular que gradualmente progride para doença em estágio terminal, quanto então necessitará de terapia renal substitutiva na forma de diálise ou transplante renal.
(a) Critério de Inclusão: Toda população de transplantados renais do quatro centros brasileiros acima de 18 anos;
(B) Critério de Exclusão: Menores de 18 anos; Transplantados renais com uso de outra combinação de imunossupressores que não sejam Tacrolimos/Micofenolato ou Tacrolimos/imTOR;
METODOLOGIA: Trata-se de um estudo retrospectivo de transplantados renais realizados no Hospital das Clínicas de Botucatu e mais três centros brasileiros nos últimos dez anos, comparando dois regimes de Imunossupressão . Os dados serão coletados via sistema de prontuários do HC UNESP e de forma colaborativa dos demais centros envolvidos.

PARECER CONSUBSTANCIADO DO CEP

DADOS DO PROJETO DE PESQUISA

Título da Pesquisa: Função renal durante 12 meses de transplante comparando tacrolimo e micofenolato com
tacrolimo e imTOR em doadores com diferentes faixas de KDPI. Um estudo retrospectivo
utilizando escore de propensão.

Pesquisador: ARLISSON MACEDO RODRIGUES

Área Temática:

Versão: 3

CAAE: 23843519.1.0000.5411

Instituição Proponente: Departamento de Clínica Médica

Patrocinador Principal: Financiamento Próprio

DADOS DO PARECER

Número do Parecer: 3.798.291

Apresentação do Projeto:

As informações descritas nos campos “Apresentação do Projeto”, “Objetivo da Pesquisa” e “Avaliação dos Riscos e Benefícios” foram retiradas dos documentos e arquivo - Informações Básicas da Pesquisa.

A doença renal crônica (DRC) é uma condição relacionada a um risco aumentado de internação hospitalar, morbidade e mortalidade por doença cardiovascular que gradualmente progride para doença em estágio terminal, quanto então necessitará de terapia renal substitutiva na forma de diálise ou transplante renal.

(a) Critério de Inclusão: Toda população de transplantados renais do quatro centros brasileiros acima de 18 anos;

(B) Critério de Exclusão: Menores de 18 anos; Transplantados renais com uso de outra combinação de imunossupressores que não sejam Tacrolimos/Micofenolato ou Tacrolimos/imTOR;

METODOLOGIA: Trata-se de um estudo retrospectivo de transplantados renais realizados no Hospital das Clínicas de Botucatu e mais três centros brasileiros nos últimos dez anos, comparando dois regimes de Imunossupressão . Os dados serão coletados via sistema de prontuários do HC UNESP e de forma colaborativa dos demais centros envolvidos.

Objetivo da Pesquisa:

(1) Comparar o regime contendo tacrolimo associado a micofenolato com tacrolimo associado a imTOR na função renal durante um ano subdividindo a amostra em faixas de KDPI. (2) Avaliar a sobrevida do paciente, sobrevida do enxerto e taxas de rejeição aguda comprovada por biópsia entre os diferentes regimes de imunossupressão.

Avaliação dos Riscos e Benefícios:

Há riscos mínimos visto ser um estudo retrospectivo colaborativo sem identificação de nome dos pacientes.

Benefícios: Demonstrar que o grupo contendo imTOR tem função renal inferior após 12 meses de transplante, auxiliando na realização de protocolos mais adequados de imunossupressão em transplantados renais.

Comentários e Considerações sobre a Pesquisa:

Trata-se de um projeto de pesquisa de Doutorado em "Fisiopatologia em Clínica Médica" do Programa de Pós-Graduação da Faculdade de Medicina de Botucatu da UNESP, orçado em R$ 450,00 com financiamento próprio e que investigará 900 pacientes a partir de seus prontuários. O projeto é bem escrito trará contribuições acadêmica em compreender a função renal durante 12 meses de transplante comparando diferentes regimes de imunossupressão em doadores com diferentes faixas de KDPI, isto é, "Kidney donor profile index" (KDPI), uma ferramenta que avalia características múltiplas de doadores a qualpermite calcular o perfil de um enxerto renal e fornece uma estimativa do resultado pós transplante.

Considerações sobre os Termos de apresentação obrigatória:

Os temos obrigatórios foram apresentados. Os pesquisadores apresentaram TCLE, adequado conforme Resolução 466 de 2012.

Recomendações:

Apresentar relatório final de atividades após término da pesquisa.

Conclusões ou Pendências e Lista de Inadequações:

Após análise em REUNIÃO EXTRAORDINÁRIA, o Colegiado deliberou APROVADO o projeto de pesquisa apresentado.

Considerações Finais a critério do CEP:

Conforme deliberação do Colegiado, em REUNIÃO EXTRAORDINÁRIA do Comitê de Ética em Pesquisa FMB/UNESP, realizada em 17/12/2019, o Projeto de Pesquisa encontra-se APROVADO.

O pesquisador deverá iniciar a coleta de dados após aprovação do CEP.
Após finalização da pesquisa, o pesquisador deverá apresentar relatório final de atividades.
Att CEP – FMB

Este parecer foi elaborado baseado nos documentos abaixo relacionados:

Tipo Documento	Arquivo	Postagem	Autor	Situação
Informações Básicas do Projeto	PB_INFORMAÇÕES_BÁSICAS_DO_PROJETO_1407848.pdf	07/12/2019 19:17:20		Aceito
Outros	Carta_resposta.odt	07/12/2019 19:10:23	ARLISSON MACEDO	Aceito
TCLE / Termos de Assentimento / Justificativa de Ausência	Termo_de_consentimento.odt	07/12/2019 19:01:59	ARLISSON MACEDO RODRIGUES	Aceito
Outros	Declaracao_instituicao.pdf	16/09/2019 18:40:54	ARLISSON MACEDO	Aceito
Outros	Analise_viabilidade.pdf	16/09/2019 18:38:33	ARLISSON MACEDO	Aceito
Outros	Anuencia_institucional.pdf	16/09/2019 18:36:20	ARLISSON MACEDO	Aceito
Outros	Anuencia_chefiaimediata.pdf	16/09/2019 18:34:54	ARLISSON MACEDO	Aceito
Projeto Detalhado / Brochura Investigador	Projeto.pdf	16/09/2019 18:30:27	ARLISSON MACEDO RODRIGUES	Aceito
Folha de Rosto	FolhaderostoPB.pdf	16/09/2019 18:28:32	ARLISSON MACEDO	Aceito

Situação do Parecer:
Aprovado

Necessita Apreciação da CONEP:
Não

BOTUCATU, 11 de Janeiro de 2020

Assinado por:
SILVANA ANDREA MOLINA LIMA
(Coordenador(a))

Appendix B

INFORMED CONSENT FORM

You are being invited to voluntarily participate in the doctoral research "Kidney function in 12 months of kidney transplantation comparing tacrolimus and mycophenolate with tacrolimus and mTORi in donors with different KDPI ranges. A multicenter cohort study using a propensity score", by Arlisson Macedo Rodrigues, student of the Graduate Program in Pathophysiology in Internal Medicine at the University of São Paulo State (UNESP), supervised by Prof. doctor Luis Gustavo Modelli de Andrade.

You were selected to participate in this research because you were part of a list of patients who underwent kidney transplantation in one of the following participating hospitals: Hospital das Clínicas de Botucatu (Botucatu-SP), Hospital Geral de Fortaleza (Fortaleza-CE), Santa Casa de Juiz de Fora (Juiz de Fora-MG) and Hospital do Rim (São Paulo-SP).

The study intends to compare the immunosuppressive treatment schemes performed in kidney transplant patients. Data from medical records will be analyzed to evaluate the drug regimen and associated characteristics during the first year of follow-up.

The purpose of this work is to provide scientific advances in choosing the best immunosuppression scheme for each group of patients, and your participation will be cooperating with the objectives of the study.

I ask for your consent to consult your medical record to collect information contained therein, such as demographic data (age, sex, race), disease history, data related to kidney transplantation, medications in use and test results.

Participation in research poses no anticipated health risks. However, if you do not want to participate, it is your right and this will not interfere with your treatment. You may withdraw your consent at any stage of the research without any prejudice to your routine follow-up.

Total confidentiality of your name, personal information, clinical and laboratory tests in relation to the data reported in this research is guaranteed.

You will receive a copy of this term and another copy will be kept on file by the researcher for five years. If you have any questions regarding your rights as a participant in this research, you can contact the Comitê de Ética em Pesquisa (CEP) through the telephone numbers: (14) 3880-1608 / 1609.

I am available if you want to talk to me during business hours, by phone (14) 3811-6105 or email arlisson_mac5@hotmail.com

I AGREE TO PARTICIPATE IN THE RESEARCH

Name:____

Signature:____

Date: ____/____/____

Responsible researcher: Arlisson Macedo Rodrigues. Dialysis Unit-HC FMB-Unesp. Rubião Junior, S/N. Phone: (14) 38116005. E-mail: arlisson_mac5@hotmail.com

Responsible supervisor: Luis Gustavo Modelli de Andrade. Dialysis Unit-HC FMB-Unesp. Rubião Júnior, S/N. Fone: (14) 3811-6005. E-mail: gustavo.modelli@unesp.br
